# A Cross-Sectional Study to Assess the Perceived Oral Health Care Needs, Barriers to Accessing Oral Health Care Services, and Utility Among the Rural Population in Chengalpattu District, Tamil Nadu

**DOI:** 10.7759/cureus.65596

**Published:** 2024-07-28

**Authors:** Varsha M. R., Sibyl Siluvai, Indumathi K. P., Divya V., Rajakumar S., Saravanan A.V.

**Affiliations:** 1 Public Health Dentistry, SRM Kattankulathur Dental College and Hospital, SRM Institute of Science and Technology (SRMIST), Chengalpattu, IND; 2 Oral Medicine and Radiology, SRM Kattankulathur Dental College and Hospital, SRM Institute of Science and Technology (SRMIST), Chengalpattu, IND; 3 Pediatric and Preventive Dentistry, SRM Kattankulathur Dental College and Hospital, SRM Institute of Science and Technology (SRMIST), Chengalpattu, IND; 4 Periodontology, SRM Kattankulathur Dental College and Hospital, SRM Institute of Science and Technology (SRMIST), Chengalpattu, IND

**Keywords:** dental utilisation, rural, perceived oral healthcare needs, dental services, barriers

## Abstract

Introduction

Utilization is the actual attendance of people at oral healthcare facilities to receive treatment. This study aimed to determine the perceived oral health care needs and barriers to utilizing dental services among rural populations.

Materials and methods

A cross-sectional survey using a questionnaire was designed to identify the perceived oral health care needs and obstacles to accessing dental facilities and their utility among rural populations. The sample size was set at 570. A data collection sheet was used to collect the required data, which included informed consent, demographic details, and the questionnaire.A pretested and validated questionnaire was used in the study.

Result

Among the 570 respondents, 43.3% of the population had experienced toothache, out of which 67.6% perceived the need for dental care. Only around 37.5% reported that their dental needs were met during the past year. Dental expenses were significantly related to access to the dentist.

Conclusion

This survey revealed that the majority of the population tends to utilize the available services only when absolutely necessary. High dental service costs and inadequate knowledge about oral problems act as major barriers.

## Introduction

Oral health plays a vital role in overall health, well-being, and quality of life [1-2]. Like other parts of the body, the mouth is teeming with bacteria, most of which are harmless. However, some bacteria can cause illness because the mouth is the doorway to your digestive and respiratory systems. Oral cancer, oral mucosal diseases, tooth loss, dental caries, and periodontal disease are all major global public health problems [1]. Oral health issues can have a variety of effects on one's quality of life. Children with poor oral health may be unable to communicate good feelings, which can have an impact on their social connections and self-esteem. Adults with poor periodontal health may be unable to express happy feelings, which can influence their self-esteem and social connections [3-4]. The prevalence and severity of oral diseases vary widely around the world and even within the same nation or region [5-6]. In terms of developing oral healthcare, several constraints arise when it comes to the rural population [4-7]. Due to various access barriers, people living in rural areas are often unable to enjoy the same benefits that urban inhabitants do, whether they are related to education, healthcare, or any other sector. Although access to dental treatment is one of the major concerns, insufficient use of the available facilities is another significant issue that hinders rural India's ability to improve oral health [4-8]. Perceived oral health refers to an individual’s view of their oral health [9]. Knowing the perceived healthcare needs of the population is essential for better planning and designing healthcare delivery [10-11].

Utilization is the actual attendance of people at oral healthcare facilities to receive treatment [12]. Rural populations encounter various barriers to the utilization of dental services, such as service costs, a lack of time, and a lack of knowledge regarding accessibility and availability [13]. In addition, inadequate perceived need for care and dental anxiety also have a substantial impact on the utilization of oral health care services. These hurdles can be overcome by encouraging people and educating them about oral health issues that alleviate fear, enabling them to adopt a positive attitude toward dental care [1].

Accurate planning of oral health care service delivery can be aided by understanding the various societal viewpoints on preference in terms of prevention or treatment, payment options for dental fees, and the preferred gender of dentists [10]. The Chengalpattu district is situated in the state of Tamil Nadu, India, and comprises many villages. This study aimed to assess the perceived oral health care needs and the barriers to accessing oral health care services and utility among the rural population in Chengalpattu district, Tamil Nadu. The objectives of the study were to identify the perceived oral healthcare needs among the rural population, determine the obstacles to obtaining dental services, and assess the service preferences among the rural population.

## Materials and methods

A cross-sectional survey was conducted using a questionnaire designed to identify the perceived oral health care needs and barriers to accessing dental care and utility among the rural population in Chengalpattu district, Tamil Nadu.

Sample size calculation

The formula 4pq/d2 was used to calculate the sample size. Substituting the P as 67.8 [14], the minimum sample size required was estimated to be 546. Considering the dropouts, the final sample size was set at 570. The sampling technique used was stratified random sampling.

Individuals over 18 years of age were included in the study. The exclusion criteria were those individuals with cognitive impairment and participants who had severe medically compromised conditions. A detailed research proposal describing the study and the proposed methodology was submitted to the ethical committee, and clearance was given by the institutional ethical committee with IEC no. SRMIEC-ST0722-40.

Questionnaire

The study involved a self-administered, pretested, and validated questionnaire. The advantage of using this questionnaire for the study was its use of both qualitative and quantitative validation methods [15]. The questionnaire was also translated into the local language (Tamil) and linguistically adapted to the study setting by using a back translation method.The conceptual equivalence between the original instruments and the back-translated versions was compared. To check the accuracy of the Tamil translation of the questionnaire, correlation between the responses in the English and Tamil versions was done. The Pearson’s correlation was found to be 0.8 (p < 0.05). The Cronbach’s level for internal consistency was 0.83. The questionnaire was pilot-tested and adjusted accordingly before being used in the main study. To test the reliability, the correlation between two sets of observations obtained at an interval of one month was calculated using a test-retest (intraclass correlation coefficient), and the correlation coefficient showed good stability (0.81).

A data collection sheet was used to collect the required data, which included informed consent, demographic details, and the questionnaire. The questionnaire consisted of three domains. The first domain was to identify the perceived oral health care needs; the second domain was to identify the barriers to obtaining oral health care needs; and the third was to assess the service preferences.

Statistical analysis

Data was entered into Microsoft Excel (Microsoft® Corp., Redmond, WA) and analyzed using IBM SPSS Statistics for Windows, Version 20 (IBM Corp., Armonk, NY). Descriptive statistics were calculated in percentages, and the Chi-square test was used to find the associations. The five-point Likert scale with scores of strongly disagree (0), somewhat disagree (1), no idea (2), somewhat agree (3), and strongly agree (4) was used for assessing the barriers to receiving dental care. The p-value was set at less than or equal to 0.05 for statistical significance.

## Results

Responses were collected from 570 study subjects, out of which 322 (56.5%) were females and 248 (43.5%) were males. The response rate was found to be 100%. The perceived oral healthcare needs of the population were found to be statistically significant (Table 1).

**Table 1:** Distribution of perceived oral health care needs of the population *Statistically significant results. p-value for statistical significance is set at less than or equal to 0.05. N: frequency distribution.

Question no.	Type of oral problem	Frequency (percentage)			
		Had the problem in the past year		Visited dentist for treatment	
		No	Yes	No	Yes
		N(%)	N(%)	N(%)	N(%)
1	Tooth sensitivity to heat, cold, sweets	359(63.0)	211(37.0)	114(54.0)	97(46.0)
2	Tooth decay or tooth cavitation	283(49.6)	287(50.3)	114(39.7)	173(60.2)
3	Bad breath	459(80.5)	111(19.5)	74(66.7)	37(33.3)
4	Defective tooth fillings or crowns	433(76.0)	137(24.0)	48(35.0)	89(65.0)
5	Inappropriate and loose dentures	550(96.5)	20(3.5)	9(45.0)	11(55.0)
6	Trauma or fracture to natural or artificial tooth	500(87.7)	70(12.3)	43(61.4)	27(38.6)
7	Tooth mobility	477(83.7)	93(16.3)	53(57.0)	40(43.0)
8	Toothache	323(56.7)	247(43.3)	80(32.4)	167(67.6)
9	Problems in tooth appearance: size, colour, space, alignment	377(66.1)	193(33.8)	85(44.0)	108(55.9)
10	Gum problems	432(75.8)	138(24.2)	86(62.3)	52(37.7)
11	Space due to missing teeth	433(76.0)	137(24.0)	88(62.2)	49(35.8)
	P-value (chi-square)	0.042* (0.325)	0.036* (0.412)	0.021* (0.285)	0.011* (0.213)

Among 570 subjects, 241 (37.5%) people responded that their dental needs were met in the past year, and 356 (62.5%) individuals responded that their dental needs were not met in the past year. Major barriers to accessing oral health care services include the high cost of service 205 (35.9%), not feeling the need 190 (33.3%), lack of time 183 (32.10%), and fear of dental procedure 175 (30.7%), which is depicted in Table 2.

**Table 2:** Distribution of barriers in accessing oral health care needs among the subjects *Statistically significant results. p-value for statistical significance is set at less than or equal to 0.05. N: frequency distribution.

Barriers	Frequency (%)				
	Strongly disagree	Somewhat disagree	No Idea	Somewhat agree	Strongly agree
	N(%)	N(%)	N(%)	N(%)	N(%)
High cost of service	52(9.1)	49(8.6)	71(33.9)	113(19.8)	92(16.1)
Fear of dental procedure	94(16.5)	49(8.6)	59(10.4)	92(16.1)	83(14.6)
Fear of infection transmission during a dental visit	112(19.6)	49(8.6)	101(17.7)	58(10.2)	54(9.5)
Not having enough time to go to the dentist	84(14.7)	50(8.8)	58(10.2)	119(20.9)	64(11.2)
I don’t feel the need	61(10.7)	49(8.6)	78(13.7)	110(19.3)	80(14.0)
I don’t care to go to the dentist	94(16.5)	61(10.7)	107(18.8)	70(12.3)	43(7.5)
P-value	6.33 ± 1.84 (0.047)*	6.82 ± 1.00 (0.310)	10.45 ± 1.65 (0.020)*	7.82 ± 1.05 (0.011)*	5.91± 1.94 (0.041)*

**Figure 1:**
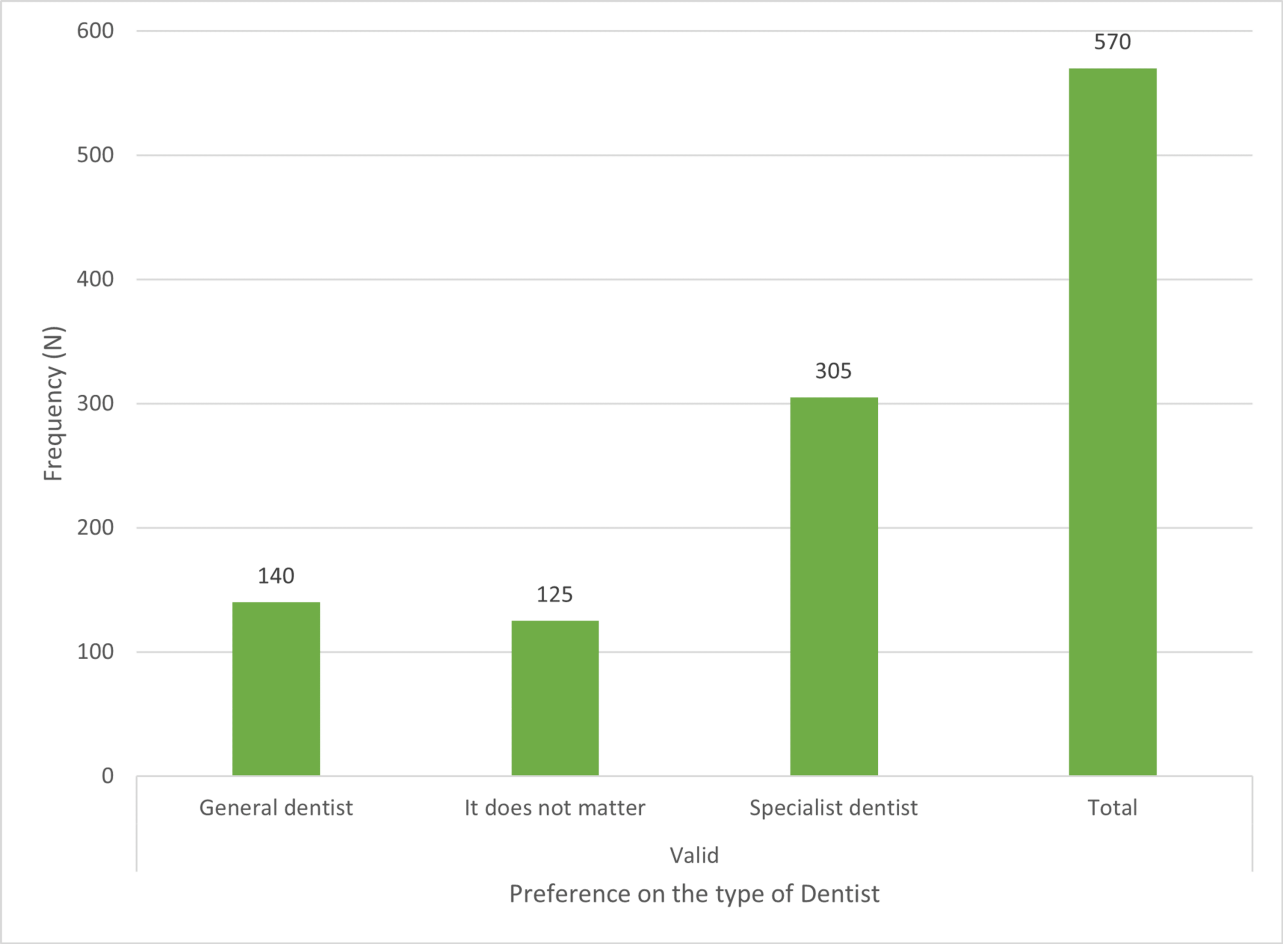
Distribution of subjects based on their preference between general or specialist dentist X-axis depicts the preference on the type of dentist. Y-axis denotes N (frequency distribution).

**Figure 2:**
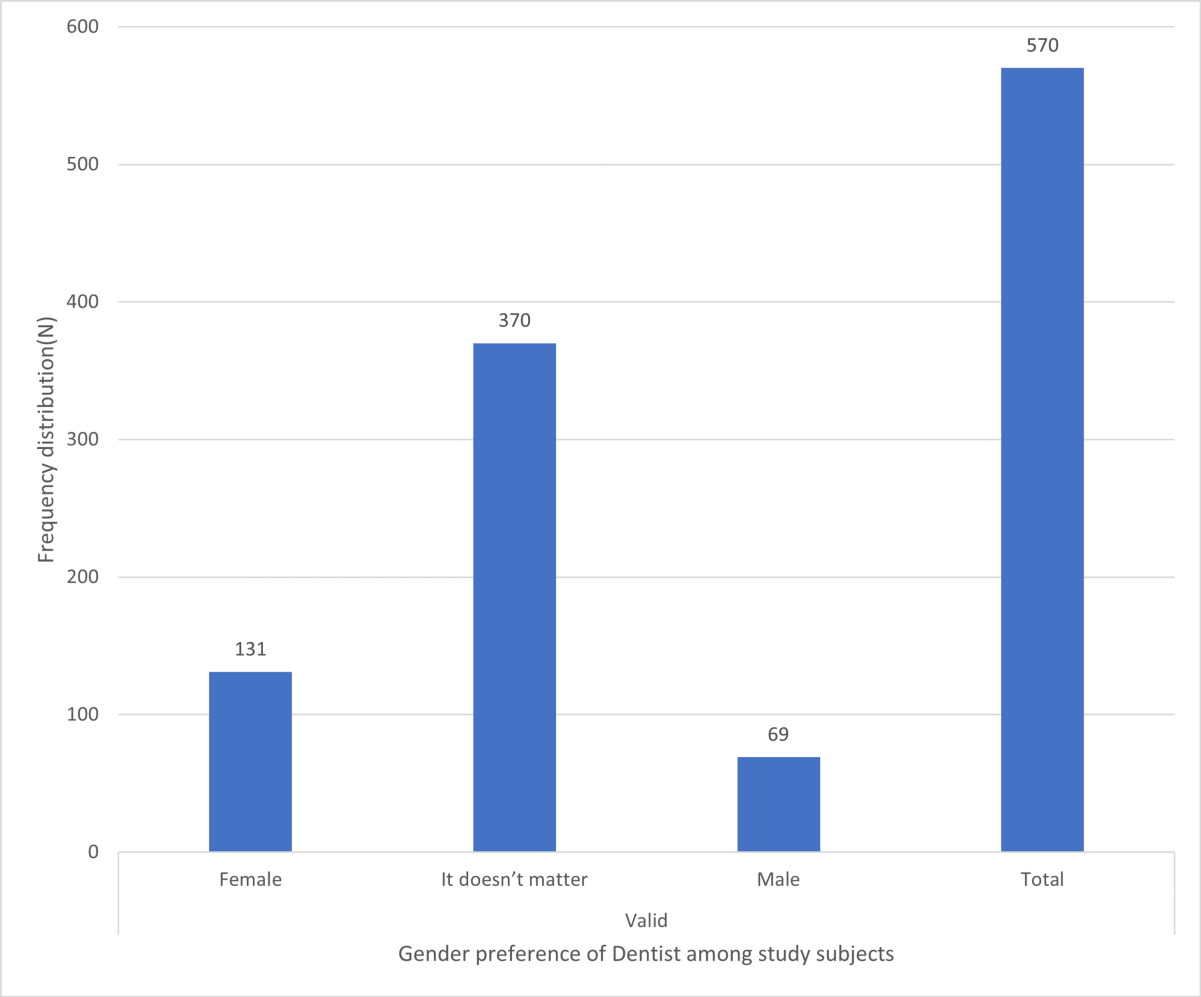
Distribution of subjects based on their preference between a male or female dentist X-axis depicts the gender preference of dentist among study subjects. Y-axis denotes N (frequency distribution).

**Figure 3:**
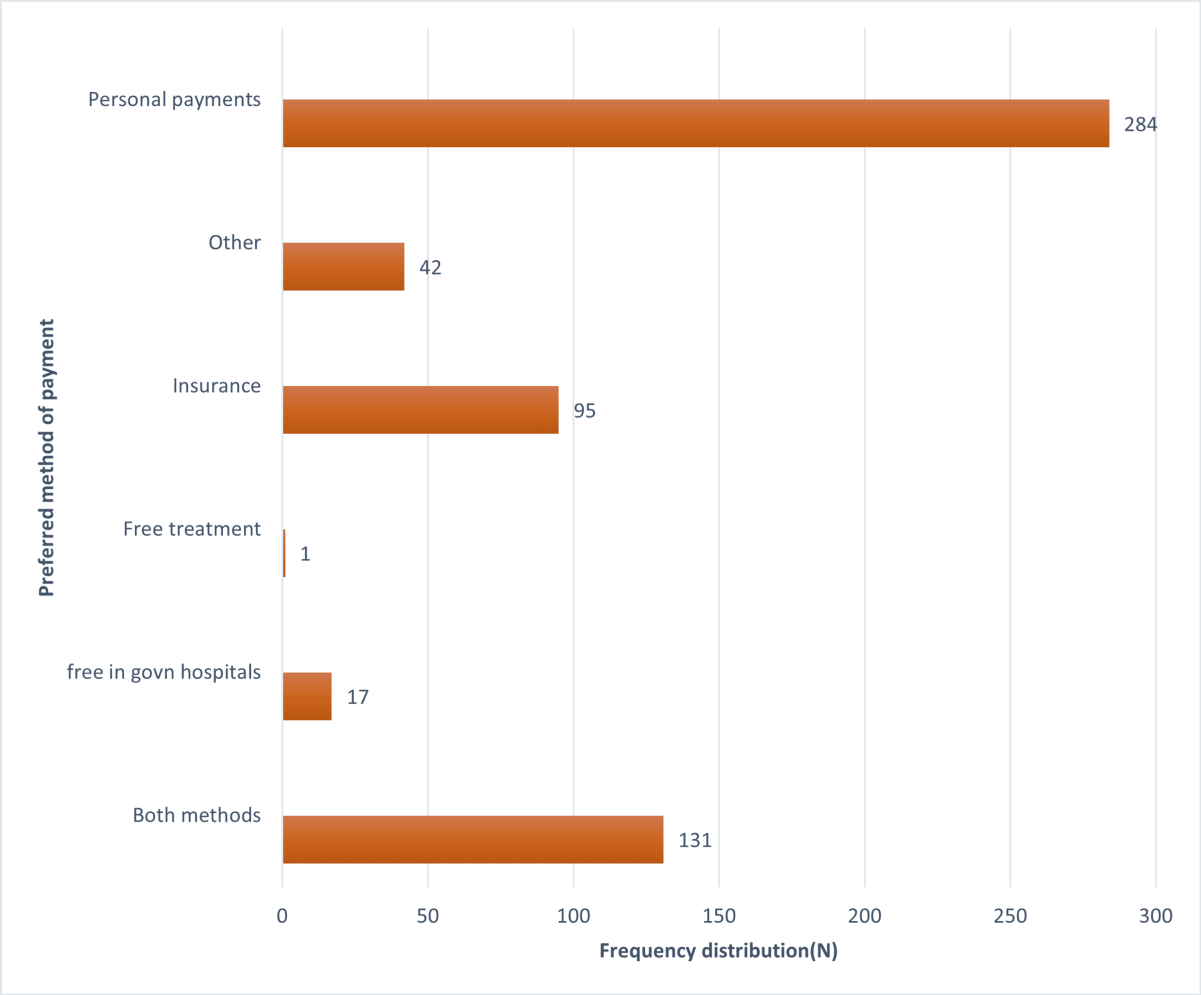
Distribution of subjects based on how they want to pay the dental fees X-axis denotes N (frequency distribution). Y-axis denotes the preferred method of payment.

Preferences among general or specialist dentists, male or female dentists, and convenient modes of paying dental fees are shown in Figures 1-3, respectively.

## Discussion

Dental issues pose a substantial risk to public health and significantly lower the quality of life, which in turn impacts everyday activities and overall well-being [16]. The study showed that 50.3% (N = 287) of the population experienced tooth decay, of which 60.2% (N = 173) received treatment for tooth decay. 43.3% (N = 173) of the population had experienced toothache, of which 67.6% (N = 167) perceived the need for dental care. Painful decayed teeth were the most common cause for people to utilize dental services. These findings are in line with the study conducted by Yaddanapalli et al. [17], which stated that decayed teeth without pain are a major factor in not utilizing dental services.

Around 37.7% (N = 52) reported that their dental needs were met during the past year. This might be because people do not visit the dentist unless they experience serious symptoms, and dentistry is seen as a beneficial service used when necessary rather than as an essential component of general health [17].

The study revealed a substantial relationship between dental service expenses and access to dentistry. This finding is in agreement with previous studies conducted by Neha et al. [16], Salim et al. [1], Hemani A et al. [18], and Bhuvaneshwari et al. [14] that showed high service costs as a predominant deterrent for regular dental visits. About 33.3% (N = 37) of the population responded that they did not seek oral care as they did not feel the need. This finding is similar to a study done by Garcha et al. [19], and this could be because dental problems are not thought to be life-threatening as compared to general health problems [17], and dental care is sought only when the condition worsens [1].

In the present study, 53.5% (N = 305) of the participants preferred a specialist dentist over a general dentist (Figure 1). 35% (N = 48) of the population opted to both prevent and treat oral problems, and 64.9% (N = 370) of participants responded that choosing a male or female dentist does not matter. 49.8% (N = 284) of participants prefer personal payments, and 16.6% (N = 95) prefer insurance for their dental expenses (Figure 3). The magnitude of personal payments is high as compared to insurance. This is because there are not many dental insurance plans available in India, in contrast to countries like the US [20] and Australia [21] that have insurance or government support [17].

However, because our study focused only on the rural population of a particular district, the results cannot be generalized to the whole population, which may be considered a limitation of the study. The responses can change depending on the area and population.

## Conclusions

The study revealed the perceived needs of the population and the hurdles faced in accessing the services. People tend to utilize the available services when it is necessary. They are unaware of the diseases until they are diagnosed or treated. This highlights the necessity of health education and awareness programs to eliminate discrepancies in the use of dental services. This study shows high dental expenses and inadequate knowledge about oral problems as major barriers. To overcome these, it is important to educate individuals about oral health issues and the available dental treatments. This will help them adopt a healthy perspective on using dental care.

## Appendices

**Table 3:** English Questionnaire Template

Name:					
Age/sex:					
Address:					
In the past year, which of the following problems have you had? Type of oral problem		Yes(or) No		(If the answer is yes): Did you go to the dentist to get treatment for the problem? (Yes, No)	
Examination and check-up					
Tooth sensitivity to heat, cold, sweets					
Tooth decay or tooth cavitation					
Bad breath					
Defective tooth fillings or crowns					
Inappropriate and loose dentures					
Trauma or fracture to natural or artificial tooth					
Tooth mobility					
Toothache					
Problems in tooth appearance: size, colour, space, alignment					
Gum problems: dental calculus, gingival bleeding, swelling, recession					
Space due to missing teeth					
If you rate your severity of need for dental treatment, what point do you give from 0 (no need) to 10 (extreme need)? 0 1 2 3 4 5 6 7 8 9 10					
Where do you go for dental care? Private clinics b) Charity clinic c) Public health centers d) Educational clinics of universities					
During the past year, do you think your dental needs were met? Yes No					
If the answer is no, why haven’t you gone to the dentist to treat your problem?					
Reasons	Somewhat agree	Somewhat disagree	Strongly agree	Strongly disagree	No idea
High cost of service					
Fear of dental procedure					
Fear of infection transmission in dental visit					
Not having enough time to go to the dentist					
I don’t feel the need					
I don’t care to go to the dentist					
If you rate your dental fear from 0 to 10, what point do you give from 0 (no fear) to 10 (extreme fear) ? 0 1 2 3 4 5 6 7 8 9 10					
Would you prefer a general or specialist dentist? a) General dentist b) Specialist dentist c) It does not matter					
Would you prefer to prevent or treat the oral problems? a) Prevention b) Treatment c) Both					
Would you prefer a male or female dentist? a) Male b) Female c) It doesn’t matter					
How would you prefer to pay dental fees? a) Insurance b) Personal payments c) Both methods d) Other __________					

## References

[1] Salim R, Ramankutty V (2021). Barriers in utilisation of dental services among older people in South Kerala. Int J Oral Health Dent.

[2] Baiju RM, Peter E, Varghese NO, Sivaram R (2017). Oral health and quality of life: current concepts. J Clin Diagn Res.

[3] Azodo CC, Ehizele AO, Umoh A, Ojehanon PI, Akhionbare O, Okechukwu R, Igbinosa L (2010). Perceived oral health status and treatment needs of dental auxiliaries. Libyan J Med.

[4] Patel RR, Richards PS, Inglehart MR (2008). Periodontal health, quality of life, and smiling patterns--an exploration. J Periodontol.

[5] Neha Neha, Reddy LV, Verma A, Shankar R (2019). Assessment of oral health status and access barriers of patients reporting to a dental college in Lucknow. J Indian Assoc Public Health Dent.

[6] Patro BK, Ravi Kumar B, Goswami A, Mathur VP, Nongkynrih B (2008). Prevalence of dental caries among adults and elderly in an urban resettlement colony of New Delhi. Indian J Dent Res.

[7] Yadav S, Rawal G (2016). The current status of dental graduates in India. Pan Afr Med J.

[8] Bommireddy VS, Koka KM, Pachava S, Sanikommu S, Ravoori S, Chandu VC (2016). Dental service utilization: Patterns and barriers among rural elderly in Guntur district, Andhra Pradesh. J Clin Diagn Res.

[9] Deep A, Singh M, Sharma R, Singh M, Mattoo KA (2020). Perceived oral health status and treatment needs of dental students. Natl J Maxillofac Surg.

[10] Yaghoubi Z, Khajedaluee M, Mohammadi TM (2017). Introducing a valid questionnaire for assessment of perceived oral health care needs, barriers to accessing oral health care services and its utility. Int J Dent Oral Health.

[11] Petersen PE (2009). Global policy for improvement of oral health in the 21st century--implications to oral health research of World Health Assembly 2007, World Health Organization. Community Dent Oral Epidemiol.

[12] Gambhir RS, Brar P, Singh G, Sofat A, Kakar H (2013). Utilization of dental care: an Indian outlook. J Nat Sci Biol Med.

[13] Shaheen SS, Kulkarni S, Doshi D, Reddy S, Reddy P (2015). Oral health status and treatment need among institutionalized elderly in India. Indian J Dent Res.

[14] Bhuvaneshwari N G, Usha G V, Lakshminarayan N (2021). Perceived dental needs and barriers to utilization of dental services among elders in India: a cross-sectional survey. J Indian Assoc Public Health Dent.

[15] Zahid O. B, Rehman A, Jamil H, Shoaib M, Bilal M, Raza F (2021). Hurdles in the access of regular dental care among the medical and dental students of Lahore. European Journal.

[16] Gambhir RS, Gupta T (2016). Need for oral health policy in India. Ann Med Health Sci Res.

[17] Yaddanapalli SC, Parveen Sultana SK, Lodagala A, Babu PC, Ravoori S, Pachava S (2020). Oral healthcare-seeking behavior and perception of oral health and general healthcare among WHO indexed age groups in East-Coast India. J Family Med Prim Care.

[18] Hemani A, Rauf F, Noori MY, Faisal A (2017). Barriers to the access of oral health care in individuals from lower socioeconomic communities in Karachi. J Liaquat Uni Med Health Sci.

[19] Garcha V, Shetiya SH, Kakodkar P (2010). Barriers to oral health care amongst different social classes in India. Community Dent Health.

[20] Manski RJ, Macek MD, Moeller JF (2002). Private dental coverage: who has it and how does it influence dental visits and expenditures?. J Am Dent Assoc.

[21] Marshall RI, Spencer AJ (2006). Accessing oral health care in Australia. Med J Aust.

